# Who gets traumatic spinal cord injury? A Finnish tertiary trauma centre study

**DOI:** 10.3389/fneur.2025.1709012

**Published:** 2026-01-12

**Authors:** Elina Luoto, Eerika Koskinen, Tuomo Thesleff, Heikki Mäntymäki, Jaakko Långsjö, Esa Jämsen, Teemu M. Luoto

**Affiliations:** 1 Department of Neurology, Tampere University Hospital, Wellbeing Services County of Pirkanmaa, Tampere, Finland; 2 Department of Neurosurgery, Tampere University Hospital, Wellbeing Services County of Pirkanmaa, Tampere, Finland; 3 Department of Intensive Care, Tampere University Hospital, Wellbeing Services County of Pirkanmaa, Tampere University, Tampere, Finland; 4 Department of Geriatrics, Faculty of Medicine (Clinicum), Helsinki University Hospital, University of Helsinki, Helsinki, Finland; 5 Department of Neurosurgery, Tampere University Hospital, Wellbeing Services County of Pirkanmaa and Tampere University, Tampere, Finland

**Keywords:** elderly, epidemiology, fall accident, prevention, spinal cord injury, spine

## Abstract

**Study design:**

Prospective cohort study.

**Objectives:**

To characterize patients with a new traumatic spinal cord injury and their pre-injury profiles.

**Setting:**

Tampere University Hospital, Finland.

**Methods:**

Newly injured patients (*n* = 46, male = 89%, mean age = 66y) with an acute cervical or thoracic traumatic spinal cord injury (TSCI) were recruited. They were evaluated and interviewed within 72 h postinjury. Health and medication history was gathered by interview and from electronic medical records. The International Standards for Neurological Classification of Spinal Cord Injury were used to classify the neurological consequences of TSCI. Epidemiological characteristics were recorded according to the International SCI Core Data Sets.

**Results:**

The leading causes of injury were low-level falls (48%), high-level falls (26%), and transport accidents (15%). Among patients >60 years, 63% were injured by low-level falls. Tetraplegia occurred in 87% of patients >60, compared to 63% ≤ 60 years. AIS D was the most common injury grade (44%). Complete injuries were seen in 38% of younger patients and 17% of older patients. Most patients had prior medication (72%) and at least one diagnosed disease (87%), both increasing in the older group. Overweight and low physical activity were common pre-injury characteristics. Alcohol preceded injury in 37% of cases. Low-level falls mostly caused cervical injuries (96%) and the patients seemed to have more diseases, fall-risk-increasing drugs and reduced physical activity levels compared to other etiologies.

**Conclusion:**

Low-level falls, particularly in older patients, were the leading cause of TSCI, often resulting in incomplete tetraplegia. Age-specific prevention strategies, especially fall prevention for older adults, are essential.

## Introduction

Traumatic spinal cord injury (TSCI) leads to motor, sensory, and autonomic impairments, affecting multiple body systems and causing a lifelong risk of various secondary complications. It significantly affects the lives of those injured as well as their social environment. Each year, Finland records around 200 new cases of traumatic spinal cord injury (TSCI) (1), and the mortality rate is higher among affected individuals than in the general population (2).

As there is currently no cure for spinal cord injuries, preventive strategies are critical to avoid the burden on individuals and society. Traumatic spinal cord injuries impose significant economic costs on society; therefore, investing in preventive measures would be justified. Recent studies indicate a rising trend in the average age at the time of TSCI, along with an increase in the proportion of cervical injuries and those caused by falls (1, 3). Also, globally, the overall risk of injury from falls increases with age (4). Understanding the epidemiological characteristics of TSCI in even more detail is crucial not only for targeting preventive measures but also for the planning of clinical care and support services for affected individuals. To our knowledge, no previous studies have systematically assessed detailed pre-injury health information. Although the proportion of TSCI resulting from low-level falls is increasing, detailed data on the pre-injury health status of patients who sustain TSCI due to low-level falls is largely lacking. The aim of this study was to provide a more detailed understanding of who is affected by TSCI and to identify pre-injury individual characteristics in different age groups giving special consideration to the pre-injury profiles of patients whose TSCIs are caused by low-level falls.

## Methods

### Setting

During the time of this study, Finland was divided into 21 hospital districts, each responsible for organizing specialized health care within its designated area. Among these, there are five university hospital districts that support the surrounding districts and provide government-mandated, highly specialized medical care to their primary referral areas. Additionally, as of May 1st, 2011, Finnish government centralized the acute care, subacute rehabilitation and life-long follow-up of SCI patients to three university hospitals: Helsinki, Oulu, and Tampere. In Tampere University Hospital’s primary referral area, Tampere University Hospital serves as the sole tertiary referral center, offering 24/7 neurosurgery and spine surgery services to a population of approximately 490,000 inhabitants.

### Study design

SUPERSTAR (Spinal Trauma Prognostication – A Tampere Research Initiative Aiming to Individualize Treatment) study is a prospective cohort study with a broad objective of improving the prognostication of individual long-term functional outcome following cervical and thoracic TSCI. The objective was approached by comprehensive data collection, consisting of pre-injury health information, injury-related characteristics, surgical and neurointensive care parameters, neuroimaging findings, blood-based neurotrauma biomarkers, genetic factors and artificial intelligence. The neurological consequences and the medical information on SCI-related issues were assessed at three time points: within the first 72 h, at 3 months, and at 1 year postinjury. In this initial study, we report the epidemiological findings of the SUPERSTAR cohort and focus on the pre- and peri-injury data. All the data provided in this substudy are based on the initial assessment. The 3-month and 1-year clinical follow-up data are not reported.

The study subjects consist of newly injured patients with acute cervical or thoracic TSCI. Patients were enrolled from the Tampere University Hospital, Tampere, Finland. The patient enrollment period lasted 32 months (Sep 2020–Apr 2023) with a 1-year follow-up period (until Apr 2024). All consecutive acute TSCI patients were applicable to participate in the study pertaining to the following inclusion criteria: (i) cervical or thoracic TSCI, (ii) admission to Tampere University Hospital within 24 h of injury, (iii) age 18 years or older, (iv) Finnish citizenship, (v) written informed consent, and (vi) no evidence of concurrent moderate or severe traumatic brain injury (Glasgow Coma Scale Score < 13 at admission or any time after). New patients were identified through reports by on-call surgeons or by daily review (study personnel) of the patient admission lists from the emergency department, neurosurgical ward, orthopedic ward and intensive care unit. Medical records of all potential patients were evaluated and eligibility was assessed. All enrolled patients gave informed consent to participate in the study, following the Declaration of Helsinki. If the patient was unable to provide a signature, the consent was taken verbally by the study physician accompanied by an additional external witness. In this case, the consent was signed by the study physician and the witness. The Ethics Committee of Pirkanmaa Hospital District, Tampere, approved the study protocol (R18182). Potential TSCI patients were screened daily and information obtained during patient screening was used to present the characteristics of the excluded patients.

### Study variables and data collection

Pre-injury health: Health history of the patients was gathered from the electronic medical records utilizing the hospital’s records and national patient data repository (Kanta archive). The medication history was obtained using the national electronic prescription archive. Pre-injury health aspects were verified by patient interview conducted by the study physicians (E.L. and E.K.). Pre-injury diseases were categorized based on the ICD-10 coding. The use of five or more drugs (≥5) was defined as polypharmacy. Fall-risk increasing drugs (FRID) were assessed by the Screening Tool of Older Persons Prescriptions in older adults with high fall risk (STOPPFall) which included 14 medication categories (5). Frailty was assessed by the Clinical Frailty Scale (CFS); scores 1–3 are considered non-frail, a score 4 is considered pre-frail, and 5–9 are considered frail (6). Alcohol Use Disorders Identification Test (AUDIT) was used to assess alcohol consumption habits. Data of locomotion and living residence were gathered. Body Mass Index (BMI) was calculated as body mass divided by height squared, weight and height were measured during the acute hospitalization. Level of leisure-time physical activity (PA) was assessed by a questionnaire with a seven-point scale ranging from activities of daily living to competitive sports (7). The response categories were (1) no activity exceeding basic activities of daily living, (2) light walking or outdoor activity one to two times per week, (3) light walking or outdoor activity several times per week, (4) brisk physical activity causing some decree of sweating and breathlessness one to two times per week, (5) brisk physical activity causing some degree of sweating and breathlessness several times per week, (6) physical activity causing sweating and rather strong shortness of breath several times per week, and (7) competitive sports and related training. For the current analyses, the categories were combined as follow: 1 and 2 = sedentary behavior; 3 and 4 = a low level PA; 5 = a medium level of PA; and 6–7 = a high level of PA.

Injury-related characteristics: A detailed injury background was outlined by combining patient interview and pre-hospital emergency personnel reports using a structured closed-ended case report form. The International Standards for Neurological Classification of Spinal Cord Injury (8) were used to evaluate and classify the neurological consequence of TSCI within 72 h after injury. Tetraplegia and paraplegia terms refer to any impairment or loss of motor and/or sensory function in the cervical or lower segments of the spinal cord. The terms tetra/paraparesis are not used. Instead, the ASIA Impairment Scale (AIS) provides a more precise approach to description of severity (i.e., completeness) of the SCI (8). Epidemiological characteristics were collected and categorized using the International SCI Core Data Set (9). In addition, falls were divided into high-level (≥1 m; ICD-10: W00-09) and low-level (<1 m; ICD-10: W10-W17) falls.

Neuroimaging Findings: As part of routine emergency management and treatment triage, a cervical or trauma computed tomography (CT) was performed at the emergency department. When clinically feasible, spinal magnetic resonance imaging (MRI) was performed after CT scanning to characterize the extent and level of injury and also to aid in the assessment of the need for acute decompressive and/or stabilizing surgery. For this study, the spinal MRI images were interpreted by a neurosurgeon (T.L.) regarding acute traumatic medullopathy. A follow-up spine MRI was performed on enrolled patients two to 3 months after the injury as part of the study protocol. The extent of decompression was evaluated using the follow-up MRI scans.

### Statistical analysis

A descriptive overview of the entire cohort was first provided followed by stratified analysis comparing groups based on age (≤60 vs. >60 years) and injury etiology (low-level falls vs. other causes). The age distribution is based on the International SCI Core Data Set and is additionally comparable to previous Finnish epidemiological studies on TSCI. Continuous variables were presented with descriptive statistics (mean = M, standard deviation = SD, median = Md, and range). The Mann–Whitney-U-test or T-test test were used to calculate the differences between patient groups. For categorial variables, the frequencies and percentages were calculated. The differences between groups were examined by the Fisher’s exact test or chi-squared test. A *p*-value < 0.05 was considered statistically significant. SPSS version 28.0 (IBM, Armonk, NY) was used to perform all the statistical analyses.

## Results

**Figure 1:**
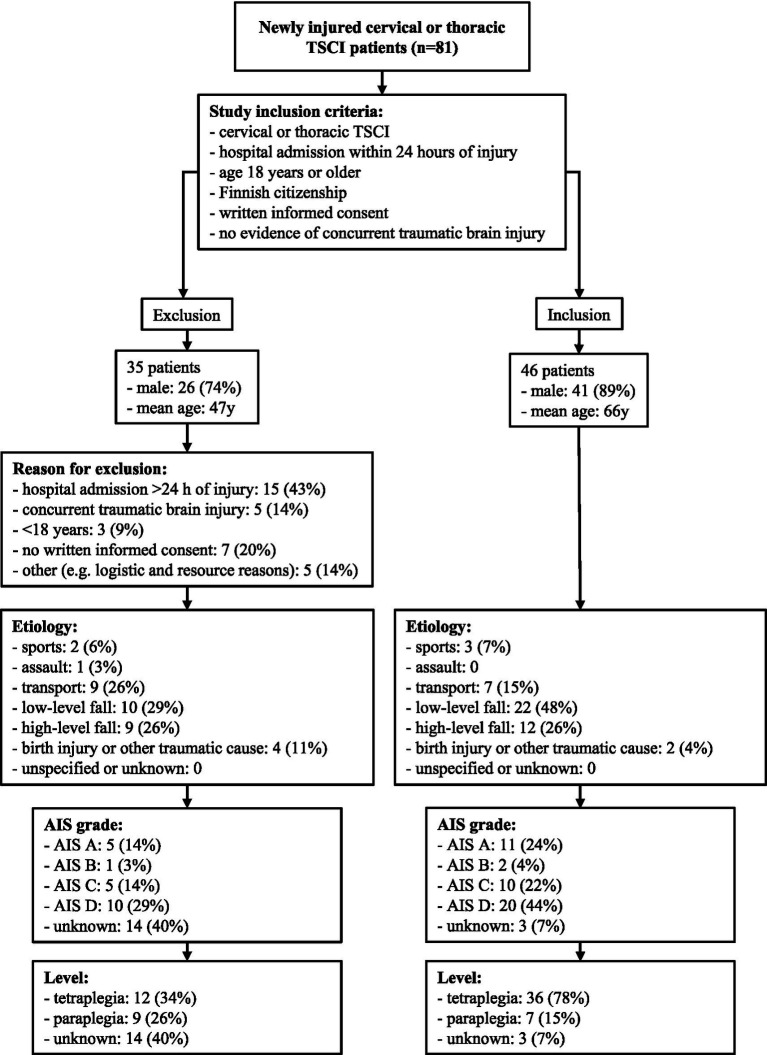
Patient enrollment process of the SUPERSTAR study.

A total of 81 cervical and thoracic TSCI patients were identified during the study period. The final enrolled cohort which met the study inclusion criteria consisted of 46 patients. A detailed overview of the inclusion process, as well as the characteristics of the inclusion and exclusion groups, is presented in Figure 1.

Injury-related characteristics are presented in Table 1. The mean age of the patients was 66 years and 89% were men. The leading cause of injury was low-level fall (48%), followed by high-level fall (26%), transport accidents (15%), sports (7%), and other traumatic causes (4%). Of the patients 78% were tetraplegic and 24% had a complete injury.

**Table 1:** Demographic and peri-injury characteristics of TSCI patients.

Variable	All (*n* = 46)	Age ≤ 60 years (*n* = 16)	Age > 60 years (*n* = 30)	*p*-value
Age at injury, Mean ± SD, Md (range)	65.9 ± 13.8 68.5(32–93)			
Gender (Male/Female)	8.2/1 (41/5)	16/0	6.0/1(30/5)	0.147
Injury etiology (%)				
Sports	3 (6.5%)	3 (18.8%)	0 (0.0%)	**0.008**
Assault	0 (0.0%)	0 (0.0%)	0 (0.0%)	
Transport	7 (15.2%)	3 (18.8%)	4 (13.3%)	
Low-level fall	22 (47.8%)	3 (18.8%)	19 (63.3%)	
High-level fall	12 (26.1)	6 (37.5%)	6 (20.0%)	
Birth injury or other traumatic cause	2 (4.3%)	1 (6.3%)	1 (3.3%)	
Unspecified or unknown	0 (0.0%)	0 (0.0%)	0 (0.0%)	
Alcohol intoxication at the time of injury (%)				
Yes	17 (37.0%)	7 (43.8%)	10 (33.3%)	0.340
No	21 (45.7%)	8 (50.0%)	13 (43.3%)	
Unknown	8 (17.4%)	1 (6.3%)	7 (23.3%)	
Vertebral injury (%)	37 (80.4%)	13 (81.3%)	24 (80.0%)	1.000
Associated injury (%)	6 (13.0%)	4 (25.0%)	2 (6.7%)	0.163
Spinal surgery (%)	38 (82.6%)	15 (93.8%)	23 (76.7%)	0.230
Hours from injury to surgery Mean ± SD, Md (range)	21.8 ± 23.2 12.0 (122) (n = 38)	24.0 ± 31.0 12.3 (122) (n = 15)	20.4 ± 17.0 12.0 (66) (n = 23)	0.436
Neurological level of injury (%)				
Tetraplegia	36 (78.3%)	10 (62.5%)	26 (86.7%)	**0.003**
Paraplegia	7 (15.2%)	6 (37.5%)	1 (3.3%)	
Unknown or not applicable	3 (6.5%)	0 (0.0%)	3 (10.0%)	
ASIA Impairment Scale (%)				
A	11 (23.9%)	6 (37.5%)	5 (16.7%)	0.419
B	2 (4.3%)	0 (0.0%)	2 (6.7%)	
C	10 (21.7%)	3 (18.8%)	7 (23.3%)	
D	20 (43.5%)	7 (43.8%)	13 (43.3%)	
E and unknown or not applicable	3 (6.5%)	0 (0.0%)	3 (10.0%)	
Neurological category (%)				
Ventilator dependent	Not applicable	Not applicable	Not applicable	0.196
C1-C4 AIS A, B, C	15 (32.6%)	4 (25.0%)	11 (36.7%)	
C5-C8 AIS A, B, C	3 (6.5%)	1 (6.3%)	2 (6.7%)	
T1-T12 AIS A, B, C	5 (10.9%)	4 (25.0%)	1 (3.3%)	
All AIS D	20 (43.5%)	7 (43.8%)	13 (43.3%)	
All AIS E	0 (0.0%)	0 (0.0%)	0 (0.0%)	
Unknown or not applicable	3 (6.5%)	0 (0.0%)	3 (10.0%)	
Days from injury to ISNCSCI Mean ± SD, Md (range)	3.6 ± 2.3 3.0 (1–11) (n = 43)	3.9 ± 2.8 3.0 (1–11) (n = 16)	3.4 ± 2.0 3.0 (1–9) (n = 27)	0.837
LOS of acute hospitalization Mean ± SD, Md (range)	15.5 ± 16.8 11.0 (1–79) (n = 43)	16.7 ± 16.7 12.5 (4–76) (n = 16)	14.8 ± 17.1 10.0 (1–79) (n = 27*)	0.186
ICU LOS Mean ± SD, Md (range)	7.1 ± 3.1 8.0 (1–16) (n = 42)	7.9 ± 3.4 8.0 (4–16) (n = 14)	6.7 ± 2.9 7.5 (1–12) (n = 28)	0.484

ISNCSCI, The International Standards for Neurological Classification of Spinal Cord Injury; LOS, length of stay; ICU, intensive care unit; * 3 patients died during the acute hospitalization. *p*-values < 0.05 are bolded.

Acute MRI was done on 91% (*n* = 42) of the cases and acute traumatic medullopathy was seen in 79% (*n* = 33) of those cases. Four of the cases were not MR-imaged because of practical reasons (e.g., instability of vitals and/or spine, and hospital logistics). In 12% (*n* = 5) of the cases, there were no neuroimaging abnormalities and in 9% (*n* = 4) of the cases it was impossible to evaluate the images (e.g., movement and metal-induced artifacts). Spinal surgery was performed in 83% (*n* = 38) of the cases. Of these, in 37 procedures a spinal cord decompression was performed, and sufficient decompression according to the follow-up MRI (2–3 months postinjury) was achieved in 29 cases (78%). Of the operated patients, surgery was performed within the first 12 h after the injury in 50% (*n* = 19) and within 24 h in 68% (*n* = 26).

Tables 2, 3 present the patients’ pre-injury background characteristics stratified by age. All but one patient lived independently in a private residence before the injury. Three patients used a stick or crutches for walking, and one used a rollator. The remaining patients were able to walk without any assistive devices. In total, 87% (*n* = 40) of patients had been diagnosed with one or more diseases prior to the injury. Multimorbidity (≥2 diseases) was more common in the older age group compared to the younger group, 87% (*n* = 26) and 63% (*n* = 10), respectively (*p* = 0.071). Diseases of the circulatory system were the most common, affecting 52% (*n* = 24) of patients, with hypertension being the most prevalent (50%). Only one patient had dementia (2%). Musculoskeletal system and connective tissue diseases were present in 46% (*n* = 21), with dorsopathies affecting 22% (*n* = 10). Prior spine surgery had been performed on 9% (*n* = 4) of the patients, and only one patient (2%) had sequelae of a previous TSCI. Of all patients, 72% (*n* = 33) were using regular medication prior to their injury. This proportion was higher in the older age group (>60 years), with 87% (*n* = 26) using medication, compared to only 44% (*n* = 7) in the younger age group (≤60 years) (*p* = 0.005). Polypharmacy was observed in 46% (*n* = 21) and at least one FRID was used by 39% (*n* = 18) of the patients. The most used medications were cardiovascular medication, particularly anti-hypertensive medication, which was used by 46% of patients.

**Table 2:** Pre-injury characteristics of TSCI patients.

Variable	All (*n* = 46)	Age ≤60 years (*n* = 16)	Age >60 years (*n* = 30)	*p*-value
BMI, Mean ± sd, Md (range)	26.9 ± 3.5 26.1 (21.7–38.9)	26.7 ± 3.4 26.0 (22.4–33.9)	27.0 ± 3.5 26.6 (21.7–38.9)	0.745
Underweight, BMI < 18.5	0 (0.0%)	0 (0.0%)	0 (0.0%)	0.825
Normal weight, BMI 18.5–24.9	14 (30.4%)	6 (37.5%)	8 (26.7%)	
Overweight, BMI 25–29.9	26 (56.5%)	8 (50.0%)	18 (60.0%)	
Obese, BMI ≥ 30	6 (13.0%)	2 (12.5%)	4 (13.3%)	
AUDIT (%)				
0–7 points	32 (69.6%)	11 (68.8%)	21 (70.0%)	**0.005**
8–13 points	3 (6.5%)	2 (12.5%)	1 (3.3%)	
≥14 points	3 (6.5%)	3 (18.8%)	0 (0.0%)	
Unknown	8 (17.4%)	0 (0.0%)	8 (26.7%)	
Clinical Frailty Scale				
Non-frail	42 (91.3%)	16 (100.0%)	26 (86.7%)	0.801
Pre-frail	1 (2.2%)	0 (0.0%)	1 (3.3%)	
Frail	1 (2.2%)	0 (0.0%)	1 (3.3%)	
Unknown	2 (4.3%)	0 (0.0%)	2 (6.7%)	
Physical activity				
1–2 Sedentary behavior	7 (15.2%)	1 (6.3%)	6 (20.0%)	0.160
3–4 Low level physical activity	14 (30.4%)	8 (50.0%)	6 (20.0%)	
5 Medium level physical activity	13 (28.3%)	3 (18.8%)	10 (33.3%)	
6–7 High level physical activity	9 (19.6%)	4 (25.0%)	5 (16.7%)	
Unknown	3 (6.5%)	0 (0.0%)	3 (10.0%)	
Number of diseases pre-injury				
0–1 diseases	10 (21.7%)	6 (37.5%)	4 (13.3%)	0.071
2–4 diseases	26 (56.5%)	9 (56.3%)	17 (56.7%)	
≥5 diseases	10 (21.7%)	1 (6.3%)	9 (30.0%)	
Polypharmacy (≥5 drugs)	21 (45.7%)	2 (12.5%)	19 (63.3%)	**<0.001**
Number of fall risk increasing drugs				
None	28 (60.9%)	13 (81.3%)	15 (50.0%)	**0.039**
Any	18 (39.1%)	3 (18.8%)	15 (50.0%)	

BMI, body mass index, AUDIT, Alcohol Use Disorders Identification Test. *p*-values < 0.05 are bolded.

**Table 3:** Pre-injury diseases and medication of TSCI patients.

Variable	All (*n* = 46)	Age ≤60 (*n* = 16)	Age >60 (*n* = 30)	*p*-value
Medical History (%)	40 (87.0%)	12 (75%)	28 (93.3%)	0.163
Neoplasms (C00-D48)	7 (15.2%)	0 (0%)	7 (23.3%)	0.078
Nervous system	2 (4.3%)	0 (0%)	2 (6.7%)	0.536
Diseases of the blood and bloodforming organs and certain disorders involving the immune mechanisms (D50-89)	3 (6.5%)	0 (0%)	3 (10%)	0.542
Anaemia (D50-64)	3 (6.5%)	0 (0%)	3 (10%)	0.542
Endocrine, nutritional and metabolic diseases (E00-90)	21 (45.7%)	5 (31.3%)	16 (53.3%)	0.152
Diabetes mellitus (E10-14)	10 (21.7%)	2 (12.5%)	8 (26.7%)	0.455
Mental and behavioral disorders (F00-99)	9 (19.6%)	4 (25%)	5 (16.7%)	0.698
Diseases of the nervous system (G00-99)	8 (17.4%)	2 (12.5%)	6 (20.0%)	0.694
Myelopathy in diseases classified elsewhere (G99.2)	0 (0.0%)	0 (0.0%)	0 (0.0%)	
Clinically diagnosed dementia	1 (2.2%)	0 (0.0%)	1 (3.3%)	1.000
Myelopathy with symptoms	0 (0.0%)	0 (0.0%)	0 (0.0%)	
Disorders of vestibular function (H81)	0 (0.0%)	0 (0%)	0(0%)	
Diseases of circulatory system (I00-99)	24 (52.2%)	6 (37.5%)	18 (60.0%)	0.146
Hypertensive disease (I10-15)	23 (50.0%)	5 (31.3%)	18 (60.0%)	0.063
Ischemic heart diseases (I20-25)	4 (8.7%)	1 (6.3%)	3 (10%)	1.000
Cerebrovascular diseases (I60-69)	2 (4.3%)	0 (0%)	2 (6.7%)	0.536
Diseases of respiratory system (J00-99)	3 (6.5%)	1 (6.3%)	2 (6.7%)	1.000
Sleep apnea (G47.3)	4 (8.7%)	1 (6.3%)	3 (10%)	1.000
Diseases of digestive system (K00-93)	8 (17.4%)	1 (6.3%)	7 (23.3%)	0.230
Sphincter dysfunction unrelated the spinal cord lesion	0 (0.0%)	0 (0.0%)	0 (0.0%)	
Diseases of musculoskeletal system and connective tissue (M00-99)	21 (45.7%)	7 (43.8%)	14 (46.7%)	0.850
Dorsopathies	10 (21.7%)	4 (25.0%)	6 (20.0%)	0.720
Spondylopathies	7 (15.2%)	3 (18.8%)	4 (13.3%)	0.681
Ankylosing spondylitis (M45)	3 (6.5%)	1 (6.3%)	2 (6.7%)	1.000
Spinal stenosis (M48)	1 (2.2%)	1 (6.3%)	0 (0.0%)	0.348
Diseases of genitourinary system (N00-99)	8 (17.4%)	1 (6.3%)	7 (23.3%)	0.230
Urinary tract impairment unrelated to spinal cord lesion	5 (10.9%)	0 (0.0%)	5 (16.7%)	0.147
Certain conditions originating in the perinatal period (P00-96)	0 (0.0%)	0 (0.0%)	0 (0.0%)	
Congenital malformations, deformations and chromosomal abnormalities (Q00-99)	0 (0.0%)	0 (0.0%)	0 (0.0%)	
Injury, poisoning and certain other consequences of external causes (S00-T98)	1 (2.2%)	0 (0.0%)	1 (3.3%)	1.000
Sequelae of intracranial injury (T90.5)	0 (0.0%)	0 (0.0%)	0 (0.0%)	
Sequelae of injury of spinal cord (T91.3)	1 (2.2%)	0 (0.0%)	1 (3.3%)	1.000
Prior surgical treatment of the brain or spine	5 (10.9%)	2 (12.5%)	3 (10.0%)	1.000
Intracranial surgery	1 (2.2%)	0 (0.0%)	1 (3.3%)	1.000
Cervical spine surgery	2 (4.3%)	1 (6.3%)	1 (3.3%)	1.000
Thoracic spine surgery	0 (0.0%)	0 (0.0%)	0 (0.0%)	
Lumbar spine surgery	2 (4.3%)	1 (6.3%)	1 (3.3%)	1.000
Prior Medication (%)	33 (71.7%)	7 (43.8%)	26 (86.7%)	**0.005**
Cardiovascular medication	23 (50.0%)	4 (25.0%)	19 (63.3%)	**0.013**
Antihypertensive medication	21 (45.7%)	4 (25.0%)	17 (56.7%)	**0.040**
Medication affecting blood clotting and anaemia	8 (17.4%)	0 (0.0%)	8 (26.7)	**0.037**
Anticoagulation	4 (8.7%)	0 (0.0%)	4 (13.3%)	0.282
Antiplatelet medication	4 (8.7%)	0 (0.0%)	4 (13.3%)	0.282
Central nervous system medication	8 (17.4%)	1 (6.3%)	7 (23.3%)	0.230
Analgesic medication	11 (23.9%)	2 (12.5%)	9 (30.0%)	0.282
Opioids	2 (4.3%)	0 (0.0%)	2 (6.7%)	0.536
Pulmonary medication	2 (4.3%)	0 (0.0%)	2 (6.7%)	0.536
Gastrointestinal medication	11 (23.9%)	1 (6.3%)	10 (33.3%)	0.068
Hormones, contraceptives and gynecologic medication	4 (8.7%)	1 (6.3%)	3 (10.0%)	1.000
Sexual and urinary organ medication	6 (13.0%)	1 (6.3%)	5 (16.7%)	0.649
Diabetes medication	8 (17.4%)	1 (6.3%)	7 (23.3%)	0.230
Cancer medication; immunosystem modulators	2 (4.3%)	1 (6.3%)	1 (3.3%)	1.000
Bone tissue medication	4 (8.7%)	0 (0.0%)	4 (13.3%)	0.282

*p*-values < 0.05 are bolded.

Low-level falls were the leading cause of TSCI. Detailed characteristics of patients stratified by etiology of low-level falls versus other etiology is presented in Table 4. Of these patients, 96% (*n* = 21) experienced tetraplegia, compared to 63% (*n* = 15) in the group with injuries caused by other etiologies (*p* = 0.009). Neurological category C1-4 AIS A, B, and C seemed to be more common in patients with low-level falls, seen in 46% (*n* = 10) of cases, versus 21% (*n* = 5) in those with injuries from other causes (*p* = 0.094). Among patients injured due to low-level falls, 18% (n = 4) had an AIS grade A injury, compared to 29% (*n* = 7) those with injuries from other causes (*p* = 0.856). None of the patients in the fall group had associated injuries, whereas 25% (*n* = 6) of those injured by other causes did (*p* = 0.022). Frailty was not prominent in either patient group. Physical activity was more commonly classified as sedentary behavior among patients injured by low-level falls (27%, n = 6) compared to those with injuries from other causes (4%, *n* = 1) (*p* = 0.160). Patients in the fall-related group also had slightly more pre-existing medical conditions and use of two or more FRIDs. There were no notable differences between the groups in terms of alcohol use or BMI.

**Table 4:** Characteristics of TSCI patients stratified by trauma etiology.

Variable	Low-level fall (*n* = 22)	Other etiology (*n* = 24)	*p*-value
Number of diseases			
0–1 disease	4 (18.2%)	6 (25.0%)	0.347
2–4 diseases	11 (50.0%)	15 (62.5%)	
≥5 diseases	7 (31.8%)	3 (12.5%)	
Polypharmacy (≥5 drugs)	10 (45.5%)	11 (45.8%)	1.000
Number of fall risk increasing drugs			
None	12 (54.5%)	16 (66.7%)	0.531
1 drug	6 (27.3%)	6 (25.0%)	
2 or more	4 (18.2%)	2 (8.3%)	
Clinical frailty scale			
Non-frail	19 (86.4%)	23 (95.8%)	0.603
Pre-frail	1 (4.5%)	0 (0%)	
Frail	1 (4.5%)	0 (0%)	
Unknown	1 (4.5%)	1 (4.2%)	
AUDIT			
0–7	17 (77.3%)	15 (62.5%)	0.469
8–13	0 (0%)	3 (12.5%)	
14–40	1 (4.5%)	2 (8.3%)	
Unknown	4 (18.2%)	4 (16.7%)	
BMI			
Normal weight	7 (31.8%)	7 (29.2%)	1.000
Overweight	12 (54.5%)	14 (58.3%)	
Obese	3 (13.6%)	3 (12.5%)	
Physical activity			
Sedentary behavior	6 (27.3%)	1 (4.2%)	0.160
Low level physical activity	4 (18.2%)	10 (41.7%)	
Medium level physical activity	6 (27.3%)	7 (29.2%)	
High level physical activity	4 (18.2%)	5 (20.8%)	
Unknown	2 (9.1%)	1 (4.2%)	
Alcohol at the time of injury			
No	10 (45.5%)	11 (45.8%)	0.308
Yes	10 (45.5%)	7 (29.2%)	
Unknown	2 (9.1%)	6 (25.0%)	
Spinal surgery			
No	3 (13.6%)	5 (20.8%)	0.702
Yes	19 (86.4%)	19 (79.2%)	
Associated injury			
No	22 (100.0%)	18 (75.0%)	**0.022**
Yes	0 (0%)	6 (25.0%)	
Injury level			
Tetraplegia	21 (95.5%)	15 (62.5%)	**0.009**
Paraplegia	0 (0%)	7 (29.2%)	
Unknown	1 (4.5%)	2 (8.3%)	
ASIA impairment scale (%)			
A	4 (18.2%)	7 (29.2%)	0.856
B	1 (4.5%)	1 (4.2%)	
C	6 (27.3%)	4 (16.7%)	
D	10 (45.5%)	10 (41.7%)	
E and unknown or not applicable	1 (4.5%)	2 (8.3%)	
Neurological category (%)			
Ventilator dependent	Not applicable	Not applicable	0.094
C1-C4 AIS A, B, C	10 (45.5%)	5 (20.8%)	
C5-C8 AIS A, B, C	1 (4.5%)	2 (8.3%)	
T1-T12 AIS A, B, C	0 (0%)	5 (20.8%)	
All AIS D	10 (45.5%)	10 (41.7%)	
All AIS E	0 (0%)	0 (0%)	
Unknown or not applicable	1 (4.5%)	2 (8.3%)	

BMI, body mass index, AUDIT, Alcohol Use Disorders Identification Test. *p*-values < 0.05 are bolded.

## Discussion

Low-level falls, especially in older adults, were the primary cause of TSCI, frequently leading to incomplete tetraplegia. Alcohol use before the injury was frequent. Overweight and physical inactivity were common among TSCI patients. Low-level falls primarily resulted in cervical injuries among older patients. These patients also had more chronic diseases at the time of TSCI, FRIDs, and reduced physical activity levels that most likely contributed to the injury risk and explain the injury mechanism.

### Epidemiological characteristics

Overall, low-level falls were the most common etiology of TSCI, particularly in the older age group, where these falls account for most injuries. This aligns with prior studies showing that older adults are more prone to both low-level falls and following fractures and TSCI (1, 3, 4, 10, 11). By contrast, the younger age group experiences a wider variety of TSCI mechanisms, with high-level falls being most common, followed by low-level falls, sports and transport accidents. Previous Nordic studies support these findings (1, 12). The diverse injury mechanisms in younger adults may reflect the higher likelihood of risky behavior.

The distribution of TSCI levels leans toward tetraplegia, particularly in the older age group. In terms of injury severity, AIS D was the most common classification across the age groups. However, younger patients were more likely to experience complete injuries (AIS A) compared to older patients. These findings are consistent with those of a previous Finnish study. This may be due to the more severe injury mechanisms commonly seen in younger adults, which are more likely to result in complete spinal cord transection. Incomplete injuries in older adults may be partially attributed to the biomechanics of low-level falls, which typically exert less force than high-impact injuries. The lack of associated injuries in patients injured by falls further reflects the difference in injury energy compared to other etiologies.

An acute spine MRI was performed in 91% of cases revealing medullopathy signal in 77% of these cases. The percentage of TSCI patients with a MRI-positive spinal cord lesions varies across studies, but a commonly cited figure is around 70–80% (13). Surgical intervention was performed on 83% of TSCI patients, with a slightly smaller proportion in the older age group. Surgical decompression within 24 h of acute TSCI is associated with improved sensorimotor recovery (14) and in our study 68% of patients were operated on within this timeframe. In neighboring countries Sweden and Norway, almost all the patients are surgically treated (96 and 89%, respectively) (12, 15). In Sweden, 61% of acute TSCI patients undergo surgery within 24 h after injury (15).

The total days required in the ICU have decreased compared to a previous Finnish study conducted between 2012 and 2016, with the mean value dropping from 9.8 days to 7.1 days. The reduction is even more pronounced in the age group ≤60 years, where the mean ICU stay decreased from 12.6 days to 7.9 days (1). The median length of stay for acute hospitalization before rehabilitation admission or direct discharge was 11.0 days, consistent with a previous Finnish study conducted between 2011 and 2015, which reported a median of 12 days (16).

### Patient characteristics

The majority of TSCI patients across age groups had at least one chronic disease documented in their medical history. This is even more pronounced in the older age group. In the older age group, a significant majority were on medication before their injury, with polypharmacy being substantially more common among older than younger patients. In contrast, medication use was rare in younger TSCI patients, likely to reflect both lower disease prevalence and reduced need for medical interventions. In line with our findings, a Japanese study, reported that most of the cervical spine injury patients had some underlying health condition, and the mean number of medications was 3.9 (17).

It is commonly accepted that alcohol increases the risk of injuries which may subsequently lead to TSCI. It is also documented that both consuming alcohol shortly before an injury and having a history of alcohol use disorder are positively correlated with incidence of TSCI (18). In our study, younger TSCI patients tended to report higher alcohol consumption habits compared to their older counterparts. This aligns with prior studies indicating a link between alcohol use and TSCI, as patients in this age group are often injured while intoxicated (1). However, we found a discrepancy between the low AUDIT scores in the older age group and the high rate of alcohol intoxication at the time of injury. This can be due to interviewer bias as patients can be unwilling to truthfully report their alcohol habits just after an intoxication-related injury. It is also important to note that even small amounts of alcohol can be harmful to older adults with multiple comorbidities and may increase the risk of falls.

The global prevalence of overweight and obesity has been increasing at an alarming rate (19). In our study, most patients with TSCI were classified as being above normal weight, with none categorized as underweight. This skew towards overweight and obesity among TSCI patients is noteworthy, aligning with previous research that shows an elevated risk of injury, particularly from falls, among overweight individuals (20). Furthermore, in the context of TSCI, obesity has been linked to less favorable rehabilitation outcomes (21).

Globally physical inactivity is rising and after 60 years of age it rises even faster (22). In terms of physical activity in our study, while sedentary behavior was more prevalent among older TSCI patients, half of the younger patients were found to engage only in low levels of physical activity. There are no previous studies on physical activity before TSCI. Results in the same direction are seen in earlier studies dealing with injuries in general (1). Although physically more active individuals have greater frequency of exposure to precipitants to injuries, less-active individuals have an increased propensity to activity-related injuries. Also, low fitness levels and physical inactivity increase the risk for walking-related falls (23). Regular physical activity reduces the incidence of non-sport and non-leisure time injuries (24).

Frailty is a multidimensional syndrome characterized by reduced physiological and cognitive reserves leading to increased vulnerability to adverse health outcomes including falls (25). To the best of our knowledge, there are only two studies that have evaluated the effect of frailty on outcomes in TSCI patients. In a recent Canadian study (26), 31% of patients over 61 years were frail. This study reported that frailty predicted adverse events, acute length of stay, and in-hospital mortality in TSCI patients. In another study with data extracted from the US Nationwide Inpatient Sample, 23% of acute TSCI patients were categorized as frail, and of these patients 77% were over the age of 65 years (27). In this inpatient study, frailty was associated with increased risk for adverse outcomes. Previously, chronological age was reported to be a factor influencing treatment decisions after TSCI and younger patients were reported to undergo surgical decompression earlier than older patients (28). Early surgical decompression is associated with greater sensorimotor recovery (14) and in this context, it would be noteworthy to consider the importance of frailty and not only age in treatment decisions. In our study, it was surprising that nearly all patients in both age groups were classified as non-frail according to the CFS. This is even in contrast with the population level prevalence of frailty. Given that frailty is a known risk factor for falls, a higher prevalence of frailty would have been anticipated specifically among patients injured by low-level falls. This clear underrepresentation of frail patients in our sample can be due to methodological limitations of the CFS. The assessment of the CFS contains a rather large degree of rater subjectivity and patients are inclined to rate their preinjury functioning too optimistically (“good-old-days” bias) (29). As a straightforward screening tool, CFS oversimplifies the complex frailty syndrome that involves multiple factors. Thus, CFS only gives a superficial insight instead of a holistic overview.

### Low-level fall

TSCI patients with low-level falls as the primary etiology exhibit distinct pre-injury characteristics that highlight the complex interplay between frailty, comorbidities, medication use, and physical activity. In Japan, patients over 65 years injured from ground-level falls tended to have poorer pre-injury health conditions, such as medical comorbidities and frailty, compared with those who fell from higher heights (17). Our research did not provide evidence to support that patients injured by low-level falls were frail prior to the injury. TSCI patients with low-level fall etiology seem to have more pre-existing diseases. Additionally, these patients have more frequent use of FRIDs. Chronic conditions and comorbidities have shown an effect on the risk of injurious falls either directly or indirectly (30). Persistent polypharmacy, particularly combined with FRIDs use, is associated with increased risk for fall injuries (30). A further compounding factor is the lower level of physical activity observed among these patients before their injury. An inactive lifestyle reduces but does not eliminate fall-prone situations. Conversely, it has been shown that physical inactivity increases the risk for walking-related falls (23).

### Strength and limitations

The strength of our study is the comprehensive and detailed data collection, prospective design and small amount of missing data among included patients. Extensive pre-injury health data gives new insight into the current characteristics of TSCI patients. The small sample size limited the ability to reliably assess some associations. The quite high percentage of excluded patients is a limitation that hinders the wider generalizability of the results.

## Conclusion

Our study of TSCI patients reveals behavioral and physical health patterns across different age groups. Overall, these findings underscore the need for age-specific prevention strategies for TSCI. For younger adults, promoting responsible alcohol use could reduce the likelihood of injury. For older adults, efforts should focus on fall prevention strategies. Efforts to optimize medication regimens, carefully managing chronic conditions and promoting physical activity could be critical in fall and TSCI prevention. Larger prospective studies focusing on low-level falls and the pre-injury vulnerability factors leading to these injuries are urgently needed. These studies would help to design effective prevention interventions to lower the burden of these injuries.

## Data Availability

The raw data supporting the conclusions of this article is available on request. All inquiries related to the data can be directed to the corresponding author.
